# Supplementary material for: Spatial pattern of perinatal mortality and its determinants in Ethiopia: Data from Ethiopian Demographic and Health Survey 2016
Source: PLoS One. 2020 Nov 23;15(11):e0242499. doi: 10.1371/journal.pone.0242499 (PMC7682862; doi:10.1371/journal.pone.0242499)
Supplement: S1 File — (DOCX) [file pone.0242499.s001.docx]

Dear PLOS ONE staff

Thank you for all information

We have organized supporting information requested one by one as follows

1. We confirm all author list and affiliations are correct on the title page of your manuscript, and that your author contributions, competing interests, and financial disclosure are correct in the document and are suitable for publication.
2. We have checked and confirmed the formatting meets PLOS ONE's typesetting requirements for References, Tables, and Figures.
3. We have checked the figure through the PACE and confirmed the figures meet plose one technical requirements.

Kind regard
