# Supplementary material for: Spatial pattern of perinatal mortality and its determinants in Ethiopia: Data from Ethiopian Demographic and Health Survey 2016
Source: PLoS One. 2020 Nov 23;15(11):e0242499. doi: 10.1371/journal.pone.0242499 (PMC7682862; doi:10.1371/journal.pone.0242499)
Supplement: Attachment — Submitted filename: 2response for editor comment (1).docx [file pone.0242499.s002.docx]

To: Natasha McDonald (Editor)

Subject: Response and incorporated Comments of manuscript

**PONE-D-20-04429**

Topic: Spatial pattern of perinatal mortality and its determinants in Ethiopia: Data from Ethiopian Demographic and Health Survey 2016.

PLOS ONE

Version 1**,** Date: June 29, 2020

**From:** Tesfaye Assebe Yadeta

We thank the editor for the constructive and important comments. We appreciate your attention to details and timely review. Based on your comments, we have improved the manuscript, our responses to comments are summarized in the table below:

| **Suggestion** | **Reply to Editor** |
| --- | --- |
| 1. Please ensure that your manuscript meets PLOS ONE's style requirements, including those for file naming. The PLOS ONE style templates can be found at | Thank you, we have corrected based on the recommended style |
| 2. We suggest you thoroughly copyedit your manuscript for language usage, spelling, and grammar. | Thank you, we have done the necessary revision as per the comments. Tara Wilfong Associate Professor MD, MPH, University Orlando, Florida Area 64 connections, Global Health Consultant for USAID Feed the Future Innovation Lab for Livestock Systems. Currently a Fulbright Scholar in Ethiopia at Haramaya University t[*wdoc@ufl.edu*](mailto:twdoc@ufl.edu)*;* [*tara.wilfong@fulbrightmail.org*](mailto:tara.wilfong@fulbrightmail.org) |
| 3. We noticed you have some minor occurrence of overlapping text with the following previous publication(s), which needs to be addressed: | We have addressed |
| 4. In your Data Availability statement, you have not specified where the minimal data set underlying the results described in your manuscript can be found. | We have specified on method part the data available free for all by request |
| 5. Please include a separate caption for each figure in your manuscript. | We separated the caption |
| 6. We note you have included a table to which you do not refer in the text of your manuscript. Please ensure that you refer to Table 3 in your text; if accepted, production will need this reference to link the reader to the Table. | Thank you, It was an error we have corrected |

To: Reviewer one

Subject: Response and incorporated Comments of manuscript

**PONE-D-20-04429**

Topic: Spatial pattern of perinatal mortality and its determinants in Ethiopia: Data from Ethiopian Demographic and Health Survey 2016.

PLOS ONE

Version 1**,** Date: June 29, 2020

**From:** Tesfaye Assebe Yadeta

We thank the reviewer for the constructive and important comments. We appreciate your attention to details and timely review. Based on your comments, we have improved the manuscript, our responses to comments are summarized in the table below:

| **Suggestion** | **Reply to reviewer one** |
| --- | --- |
| 7. Generally the English should be polished. There are many places throughout the manuscript. | Thank you, English language edited |
| 8. line 35: Be specific on the type of GIS software, is it ArcGIS?. | Thank you, we made an error and corrected |
| 9. lines 42-45: These lines is a repeat of the results section. There is no conclusion drawn here. | Thank you reviewer, we have modified as commented |
| 10. lines 63 to 67: please specify which of the citations 7 to 9 is identified for each determinant. Does it mean each of the 3 citations have all the determinants in them. | We specified the reference for each determinant |
| 11. line 72: the citation at the end of that line should be 11. | We checked and corrected |
| 12. line 154: The outcome should change to Y_ij so that i should be for the child and child-level factors, while j is for the community factors. | Thank you we have corrected per recommendation |
| 13. line 161: insert comma after rural, and before distance | Thank you we have corrected |
| 14. Tables 1 and 2: These should be changed so that the last columns should report the percentage of children who died in pre-natal (row percentages). In other words, these tables should already inform the reader on the association between the outcome and risk factors. Therefore some of the text, in lines 215 to 229, should change to reflect these patterns. | We have modified the table per recommendation |
| 15. Table 3 is not cited in the text. This should appear on lines 252 onwards. | Thank you we made correction |
| 16. Text on lines 282 to 312 should refer to a Table. | We have corrected as recommended |
| 17. Line 399 mentions ''recall bias''. How was this resolved, or how does it affect the results. | We described and cited for details |

To: Reviewer two

Subject: Response and incorporated Comments of manuscript

**PONE-D-20-04429**

Topic: Spatial pattern of perinatal mortality and its determinants in Ethiopia: Data from Ethiopian Demographic and Health Survey 2016.

PLOS ONE

Version 1**,** Date: June 28, 2020

**From:** Tesfaye Assebe Yadeta

We thank the reviewer for the constructive and important comments. We appreciate your attention to details and timely review. Based on your comments, we have improved the manuscript, our responses to comments are summarized in the table below:

| **Suggestion** | **Reply to reviewer one** |
| --- | --- |
| 18. Abstract, lines 37-41 - Can you list separately those positively and negatively associated with PMR? | Thank you, we have modified pre recommendation |
| 19. Abstract, lines 43-45 - This sentence repeats the one in the results in lines 38-41. No need to say this twice. IN the conclusion you should focus more on the implications. | Thank you, we have modified per recommendation |
| 20. Background, line 54 - This is not a very good opening sentence. How about "Perinatal mortality includes stillbirths and early neonatal deaths, and is an important indicator of...". | The sentence edited as recommended |
| 21. Background, lines 56-57 - This sentence doesn't read well. | Thank you edition made |
| 22. Background, lines 63-67 - Where do these risks relate to? Need to start the sentence by saying something like "Multi-country reviews found that ..., ... and ... were significantly associated with perinatal mortality risk.". | We have corrected per recommendation |
| 23. Methods, line 92 - Hasn't there been a more recent census? | Yes, |
| 24. Methods, line 99 - Please mention which EDHS survey this refers to before stating the number of participants. I know the dates of data collection are given below, but we still need to know which survey you are referring to before you give the numbers. | We have mentioned as recommended |
| 25. Methods, lines 146-147 - Elsewhere DHS defines a stillbirth as "Number of foetal deaths in pregnancies of seven or more months", which means the same, but is a bit more specific in relation to the foetus. | We used DHS definition |
| 26. Results, line 215 - You haven't defined the study period in the methods. Is this all pregnancies, or just in the last five years? | We have included the study period and study participant, in the last five tears |
| 27. Results, lines 216-217 - Better to give median and range for age, as likely not to be normally distributed. | Than you, we present median and range |
| 28. Tables - 1 d.p. would be sufficient, depending on journal's policy.. | We include perinatal mortality also |
| 29. Results, line 224 - Again, median and range would be better. | Than you, we analyses median and range |
| 30. Results, line 232 onwards - Before exploring the spatial distribution and factors associated with PNM, you need to present the overall PMR in this survey. Would also be good to show PMR for each variable/category in Table 2. | Thank you, we presented PMN the overall variables per recommendation using text and chart |
| 31. Results, line 258 - Could you call this post-secondary education to make it clearer? Usually this would be expressed as "83% lower", as 1 is the reference. | We have modified us commented |
| 32. Results, line 259 - Likewise, this is "37% lower". Please check with all AORs below 1 that are reported in the text. | Thank you, it was an error we have corrected, also through the document |
| 33. Table 3 - You need to explain the difference between Model 2, 3 and 4 in the headings or footnote to the table. Also where is model 1? Is that a crude model? Why not show that one as well? | We have explained per recommendation |
| 34. Table 3 – Do all models have random effects for cluster? Or only models 3 and 4? | Detail explanation made |
| 35. Table 3 – You could exclude some variables from multivariable models that are not associated with PNM in the crude model. | Since the analysis is multilevel, model one is null model |
| 36. Table 3 - May be better to combine secondary and higher education into one group, to reduce the number of parameters in models and reduce data sparsity. | Thank you we made per recommendation |
| 37. Results, line 288 - Was household-level clustering/random effects included in the model? Or these were just factors related to the household? I don't think you could include household-level clustering/random effects for PNM since there would be many households with only one pregnancy. So if it is not included as a random effect in the multi-level model, better to call these "household-related factors" to avoid confusion with levels in the model. | Yes we have included and explained detail |
| 38. Table 4 - Models 2 and 3 seem to be the opposite way around to Table 3. I think Model 2 should be individual only and model 3 should be community only. | Thank you it was an error we have corrected |
| 39. Discussion, line 353 - decreased by 41%. | It was an error we made correction |
| 40. Discussion, line 357 - Your data shows less than 15 months. If you want to show for less than 2 years you need to re-categorise this variable. | We used less than 15 months and compare with the same categories |
| 41. Discussion, lines 401-403 - I don't understand this sentence. The same person cannot have a stillbirth and a live birth in the same month unless they are twins and born at the same time. | We described and cited for details |
| 42. Figure 2 - The colour scheme in the legend is not visible. Are the points shown on the map the sampled clusters from the survey? What do the different colours of the geographical regions mean? | We modified |
| 43. 26. Figure 3 - You need to explain LLR in full, or in a footnote so that this can be interpreted as a standalone figure. Or change the legend as this is not actually showing perinatal mortality rates, but likelihood ratios. So explain what is being compared with what. | Explained as recommendation |

To: Reviewer three

Subject: Response and incorporated Comments of manuscript

**PONE-D-20-04429**

Topic: Spatial pattern of perinatal mortality and its determinants in Ethiopia: Data from Ethiopian Demographic and Health Survey 2016.

PLOS ONE

Version 1**,** Date: June 29, 2020

**From:** Tesfaye Assebe Yadeta

We thank the reviewer for the constructive and important comments. We appreciate your attention to details and timely review. Based on your comments, we have improved the manuscript, our responses to comments are summarized in the table below:

| **Suggestion** | **Reply to reviewer one** |
| --- | --- |
| 44. Line 99-100: Already pointed out above but the description of the study participants is unclear possibly an issue with English writing but equally a technical issue introducing a bias). It can read as if women who were 7 month-pregnant at the time of the survey were included in the study but I don’t think that’s what the author meant? Or is it? It is not possible to count the number of stillbirths and neonatal deaths (the numerator of perinatal mortality rate) until after all the pregnancies have ended. So I don’t think “7320 women pregnant” (assuming that this means 7320 pregnant women) represent the study subjects?   However, having now read line 168 onward, it seems that the study included “pregnant women with 7 months and more”. How do we know that the babies from these women will live or die? | Thank you, we have corrected based on the recommended style |
| 45. At line 138-139, the same information is repeated as above that 7320 women…were included. No need to repeat the same information. | Thank you, we have avoided repeat ion |
| 46. Line 107: The same information as line 88 is repeated here. | We have corrected |
| 47. Line 158: Abbreviations need to be spelt out at first appearance. | Thank you, we have performed |
| 48. It would have been more informative if the characteristics of women who had a perinatal death and who didn’t were compared, rather than describing the characteristics of the entire study subjects as in Table 1 and table 2 | We described perinatal mortality as recommendation |
| 49. Measure of variation and model fit statistics   I think the columns in table 4 need to be swapped/re-labelled. The text at line 288 reads that after adjusting the model for “household level factors (model II)”…., but in table4, I see that model 2 is adjusted for community level factors, which should actually be model 3? | Thank you we have modified |
| 50. Linen 349- Can the authors provide some explanation for the relationship between older mothers and perinatal mortality? Dismissing it as “unclear” sounds sloppy. | We have added possible explanation |
| 51. The health extension program is discussed and it is suggested that “the findings of our study serve to further emphasize the important role that HEWs must continue to play in relaying information about perinatal death” but results of the study suggest the importance of institutional delivery. What role doe the HEWs play in institutional delivery? Could the authors comment? | HEW can educate for health care utilization and we have explained |
| 52. Line 401- I don’t quite understand what the authors are trying to say ‘in case of a stillbirth and a live birh,,,’ | We explained and cited for details |
