# Supplementary material for: Spatial pattern of perinatal mortality and its determinants in Ethiopia: Data from Ethiopian Demographic and Health Survey 2016
Source: PLoS One. 2020 Nov 23;15(11):e0242499. doi: 10.1371/journal.pone.0242499 (PMC7682862; doi:10.1371/journal.pone.0242499)
Supplement: Attachment — Submitted filename: 5response for reviewer.docx [file pone.0242499.s003.docx]

To: Reviewer two

Subject: Response and incorporated Comments of manuscript

**PONE-D-20-04429**

Topic: Spatial pattern of perinatal mortality and its determinants in Ethiopia: Data from Ethiopian Demographic and Health Survey 2016.

PLOS ONE

Version 2**,** Date: October 15 2020

**From:** Tesfaye Assebe Yadeta

We thank the reviewer for the constructive and important comments. We appreciate your attention to details and timely review. Based on your comments, we have improved the manuscript, our responses to comments are summarized in the table below:

| **Suggestion** | **Reply to reviewer one** |
| --- | --- |
| 1. Abstract, line 31 – “…women who at delivered at seven or months…” | Thank you, we have edited |
| 2. Abstract, line 45 – Shouldn’t this be for longer birth intervals? | Thank you, we made an error and corrected |
| 3. Abstract, lines 47-48 – do you mean “Disparities in perinatal mortality rates should be addressed alongside efforts to address | We have edited |
| 4. Background, line 54 – is higher | Corrected |
| 5. Background, line 67 – delete “which” | Deleted |
| 6. Background, lines 71-79 – needs some copy editing | We made copy editing |
| 7. Discussion, line 349 – associated with reduced perinatal mortality | We have corrected |
| 8. Conclusion, line 443 – Again, shouldn’t this be longer birth intervals? | We made correction |
| 9. Tables 1 and 2 – the addition of the perinatal mortality is useful in these tables, but this should be expressed as the PMR for each category, not the column percent adding up to 100%. For example, we want to know the perinatal mortality rate for rural and the perinatal mortality rate for urban so that we can see which is higher. | Thank you we have made correction as recommended |

To: Reviewer three

Subject: Response and incorporated Comments of manuscript

**PONE-D-20-04429**

Topic: Spatial pattern of perinatal mortality and its determinants in Ethiopia: Data from Ethiopian Demographic and Health Survey 2016.

PLOS ONE

Version 2**,** Date: October 15, 2020

**From:** Tesfaye Assebe Yadeta

We thank the reviewer for the constructive and important comments. We appreciate your attention to details and timely review. Based on your comments, we have improved the manuscript, our responses to comments are summarized in the table below:

| **Suggestion** | **Reply to reviewer one** |
| --- | --- |
| Line 31: “7 days postpartum” is not needed. | Thank you It include early neonatal period, we left as it is |
| Line 44: Not sure if it is correct to refer to mortality as prevalence. Could you consider changing this? | Thank you, we changed as recommended |
| Line 45: Women’s education was not associated with perinatal mortality, so perhaps need to drop the mention of women’s education here. | Women education dropped as suggested |
| Line 54: Perinatal mortality ‘has’ should be changed to ‘is’ | We corrected |
| Line 67: ‘which’ can be deleted. | We have deleted |
| Line 73: I think this sentence is awkward. |  |
| Line 98: can you add ‘of whom’ just before ‘7230 women….were used…’ | We have added |
| Line 223: ‘live’ birth rather than ‘alive’ birth. | We corrected as suggestion |
| Line 225: Can you please check if the statistics for urban residence is correctly reported? I assume that 84% were rural residents rather than urban residents. I think Table 1 also needs to be corrected in relation to this. | Yes we have checked the report was correct |
| Line 233-238: It would be much better if the order of presenting different factors correspond with the order they appear in table 2. | Thank you we have made arrangement |
| Line 247: Can you please check this statistics> Mortality was higher in urban than in rural area? This is different from what is reported in the abstract and also not in line with existing literature. I suspect this is a typo. Should have been higher in rural area’? | We have checked, in the model it is correct. We present as perinatal mortality rate in table one also |
| Line 249: I think Figure 1 is currently ‘Global Moran’s I test of cluster distribution for perinatal mortality in Ethiopia, from EDHS 2016’. Please check the figures and order them correctly. | We have corrected |
| Line 271: The sentence is awkward. Can drop ‘were’? | We have dropped |
| Line 363: “A possible explanation” rather than ‘A possible the explanation” | We made correction |
